# From integrative structural biology to cell biology

**DOI:** 10.1016/j.jbc.2021.100743

**Published:** 2021-05-04

**Authors:** Andrej Sali

**Affiliations:** Research Collaboratory for Structural Bioinformatics Protein Data Bank, the Department of Bioengineering and Therapeutic Sciences, the Quantitative Biosciences Institute (QBI), and the Department of Pharmaceutical Chemistry, University of California, San Francisco, San Francisco, California, USA

**Keywords:** structural biology, integrative structural biology, integrative structure modeling, integrative modeling, cell biology, 3DEM, three-dimensional electron microscopy, IHM, Integrative/Hybrid Methods, IMP, *Integrative Modeling Platform*, NPC, Nuclear Pore Complex, PDB, Protein Data Bank, PDB-Dev, PDB-Development, SAS, small-angle scattering, wwPDB, Worldwide Protein Data Bank

## Abstract

Integrative modeling is an increasingly important tool in structural biology, providing structures by combining data from varied experimental methods and prior information. As a result, molecular architectures of large, heterogeneous, and dynamic systems, such as the ∼52-MDa Nuclear Pore Complex, can be mapped with useful accuracy, precision, and completeness. Key challenges in improving integrative modeling include expanding model representations, increasing the variety of input data and prior information, quantifying a match between input information and a model in a Bayesian fashion, inventing more efficient structural sampling, as well as developing better model validation, analysis, and visualization. In addition, two community-level challenges in integrative modeling are being addressed under the auspices of the Worldwide Protein Data Bank (wwPDB). First, the impact of integrative structures is maximized by PDB-Development, a prototype wwPDB repository for archiving, validating, visualizing, and disseminating integrative structures. Second, the scope of structural biology is expanded by linking the wwPDB resource for integrative structures with archives of data that have not been generally used for structure determination but are increasingly important for computing integrative structures, such as data from various types of mass spectrometry, spectroscopy, optical microscopy, proteomics, and genetics. To address the largest of modeling problems, a type of integrative modeling called metamodeling is being developed; metamodeling combines different types of input models as opposed to different types of data to compute an output model. Collectively, these developments will facilitate the structural biology mindset in cell biology and underpin spatiotemporal mapping of the entire cell.

## Progress of structural biology: From single molecule to cell

### Introduction

**Figure 1:**
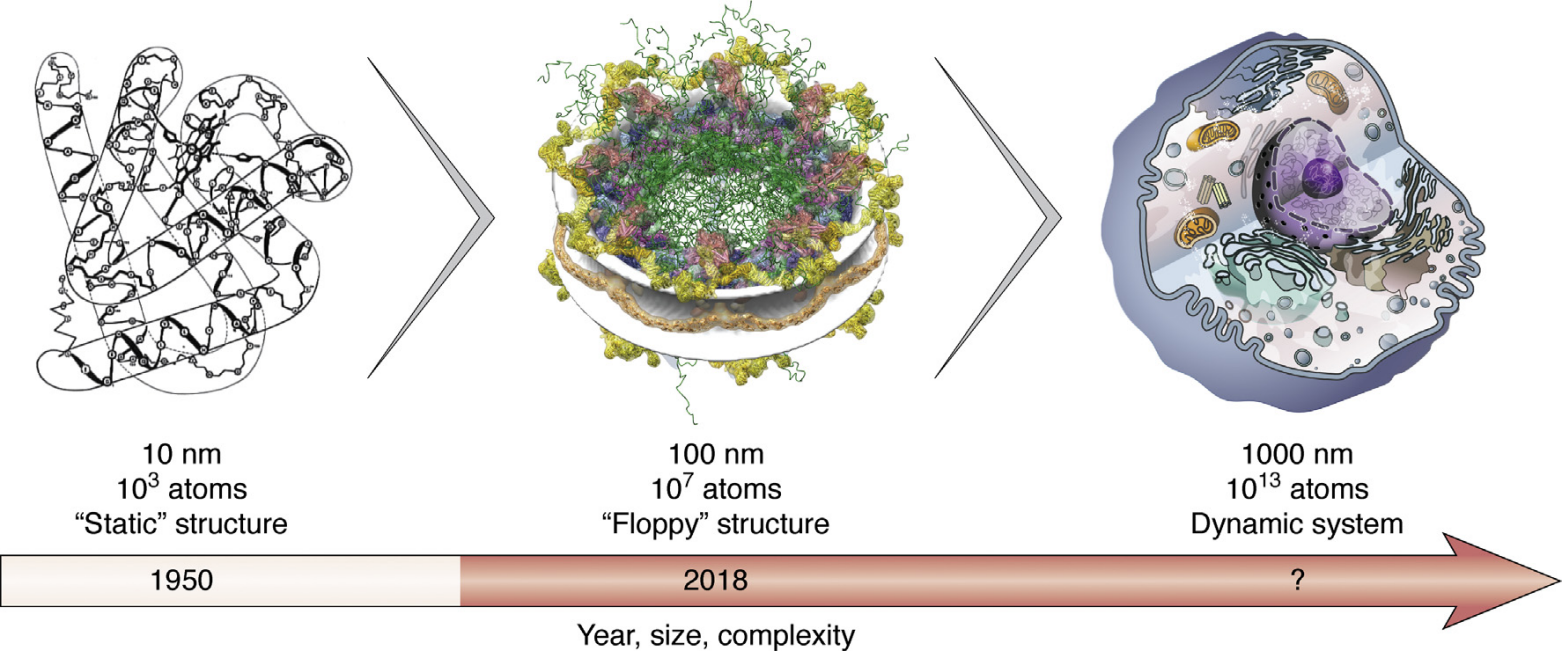
**Progress of structural biology.***Left*, the myoglobin structure determined by X-ray crystallography at atomic resolution (1, 2). *Center*, the molecular architecture of the yeast NPC determined by an integrative approach at nanometer resolution (4). *Right*, a visualization of the eukaryotic cell based on the cell biology literature (drawing by Yekaterina Kadyshevskaya). This review focuses on the role and challenges of integrative modeling of complex biomolecular systems (*red arrow*).

Structural biology was born in the 1950s, with the atomic structure of the myoglobin molecule determined by modeling based primarily on data from X-ray crystallography (1, 2) (Fig. 1). The toolbox and scope of structural biology have been expanding ever since. In steady progress, crystallography was joined by nuclear magnetic resonance (NMR) spectroscopy and 3D electron microscopy (3DEM), resulting in molecular architectures of 183,234 biomolecular systems as of February 3, 2021 (3). Some of these structurally defined systems contain as many as hundreds of protein subunits, as exemplified by the Nuclear Pore Complex (NPC) (4). Further progress toward mapping spatiotemporal organization of even larger modules of the cell, and eventually the entire cell, is inevitable. Such complex depictions are unlikely to be obtained using data from any single method. Instead, they are expected to be computed by integrative (hybrid) modeling that combines data from multiple experimental methods and prior models of parts of the entire system (5, 6).

### Outline

Here, we review the integrative approach and some of the key roles that the Worldwide Protein Data Bank (wwPDB; https://wwpdb.org) (7) has played in its development. In particular, we describe the goals and workflow of integrative structure modeling (5), illustrated by its application to determining the molecular architecture of the NPC. We then outline our open-source *Integrative Modeling Platform* (IMP) program for computing and validating integrative structure models (8, 9). Next, we discuss a number of key challenges in the further development of integrative modeling. Some of these challenges are being addressed under the auspices of the wwPDB, including the expansion of the PDB to archive and validate integrative models and the data on which they are based (10, 11). These activities illustrate the key role of the PDB in nucleating the structural biology community and expanding its scope. A particularly ambitious goal is discussed last, namely, the application of a new class of integrative modeling to the cell mapping problem, in principle fully integrating structural biology and cell biology (12, 13).

## Integrative structure modeling

### Integrative structures

Some of the very first models of biomolecular structures, including the model of the DNA double helix (14), were in fact integrative models based on a multitude of considerations. Modern integrative structure modeling is inspired by the early fitting of crystallographic subunit structures into a 3DEM density map of the actin–myosin complex (15). Many examples of recent integrative structures were reviewed (5), ranging from small proteins and nucleic acids to the entire genome.

### Definition of integrative modeling

Integrative modeling is motivated by the desire to utilize all available information about the modeled system, in an effort to maximize the accuracy, precision, and completeness of the resulting model (5, 6, 8, 10, 11, 16, 17, 18, 19, 20, 21, 22, 23, 24, 25, 26, 27, 28). A large variety of experimental and computational methods can provide input information for integrative modeling of biomolecular structures (5). Examples include X-ray crystallography, NMR spectroscopy, 3DEM, small-angle scattering (SAS), chemical cross-linking with mass spectrometry, affinity copurification, quantitative genetic interaction mapping, molecular mechanics force fields, statistical potentials, comparative protein structure modeling, and sequence covariation. Information used for integrative modeling can be at either high or low resolution (*e.g.*, nuclear Overhauser effect and affinity copurification, respectively). It can also be dense or sparse (*e.g.*, a typical X-ray diffraction dataset and an SAS profile, respectively). Given input information, integrative modeling then aims to find “all” models whose properties match the input information within an acceptable tolerance. This description in fact applies to all structure determination methods. For example, a crystallographic structure is computed by finding a set of atomic coordinates whose computed diffraction pattern in a given crystal arrangement reproduces the observed diffractions while also satisfying stereochemistry rules; a 3DEM structure is computed by finding a set of atomic coordinates whose shape matches that of the observed 3DEM map and satisfies stereochemistry; and an *ab initio* model is computed by finding a set of atomic coordinates that come as close as possible to ideal values for distances, angles, and other spatial features as specified by an energy function. The only distinction of integrative modeling is that it aims to use explicitly all available information of any type.

### Integrative modeling workflow

**Figure 2:**
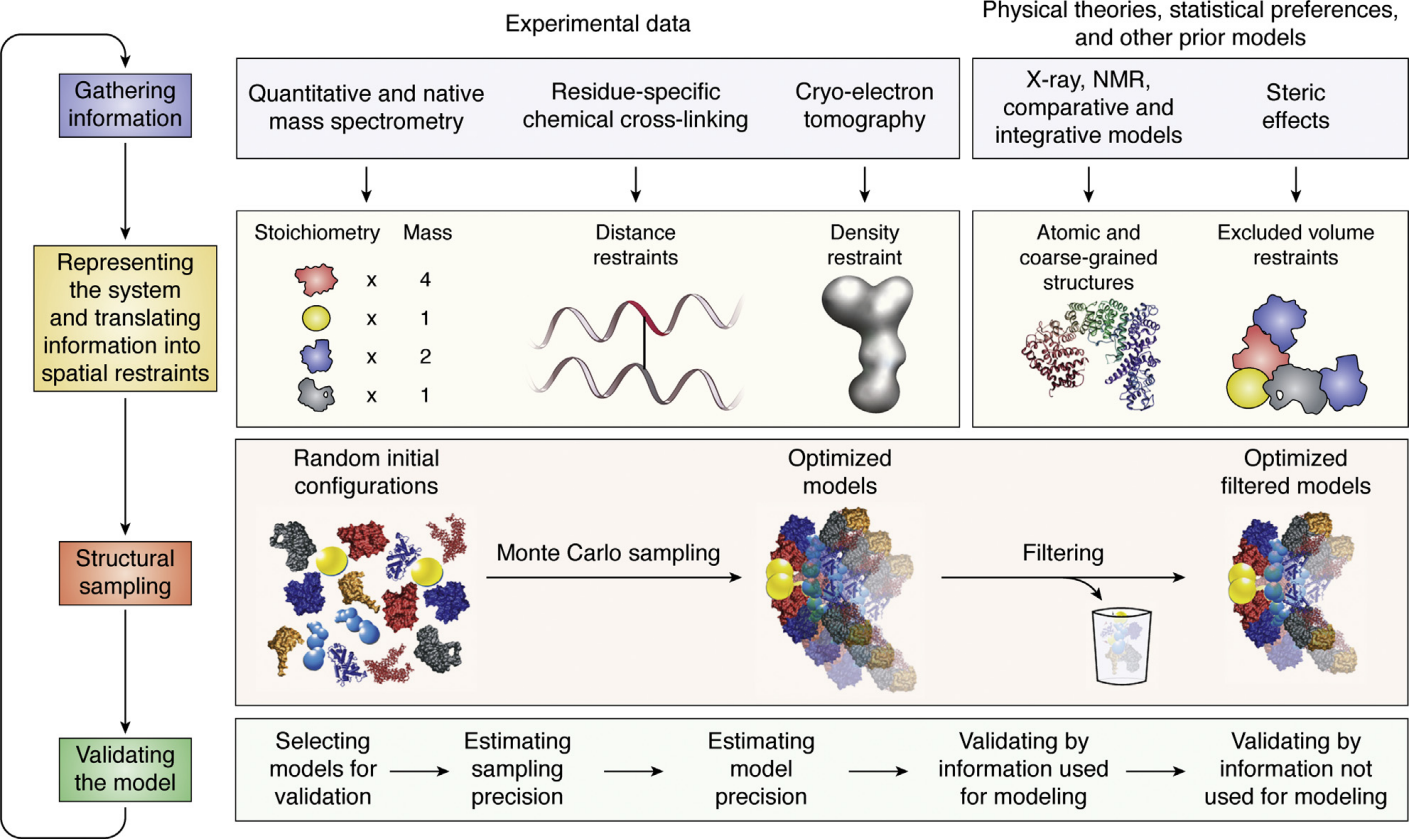
**Description of the iterative integrative modeling workflow** (5)**.** In this example, representations of the components of a complex are based on models of its components. Some component representations are coarse-grained by using spherical beads corresponding to multiple amino acid residues to reflect the lack of information and/or to increase the efficiency of structural sampling. The scoring function consists of spatial restraints that are obtained from chemical cross-linking with mass spectrometry (CX-MS) cross-links and a cryoelectron tomography density map. The sampling explores both the conformations of the components and/or their configuration, searching for those assembly structures that satisfy the spatial restraints as well as possible. The result is an ensemble of many good-scoring models that satisfy the input data within acceptable tolerances. The sampling is then assessed for its precision, followed by clustering and evaluating the models by the degree to which they satisfy the input information used to and not used to construct them. The protocol can iterate through the four stages until the models are judged to be satisfactory, most often on the basis of their precision and the degree to which they satisfy the data.

**Figure 3:**
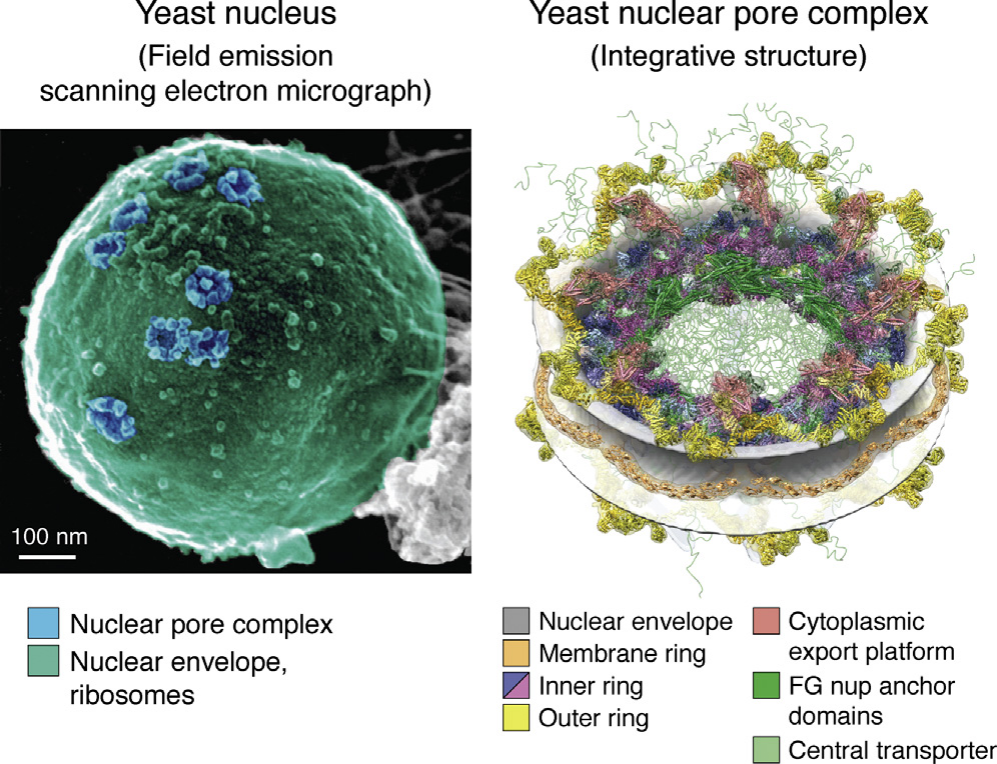
**The yeast NPC.***Left*, field emission scanning electron micrograph of the yeast nucleus (119). *Blue* pseudocoloring highlights the NPCs; *green* pseudocoloring highlights the nuclear envelope together with the attached ribosomes. Scale bar represents 100 nm. *Right*, integrative structure of the yeast NPC (4). The 8-fold symmetric modular organization of the NPC is indicated, in the context of the nuclear envelope (*gray*): membrane ring (*salmon*), inner ring (*blue* and *purple*), outer rings (*yellow*), cytoplasmic export platform (*red*), FG nup anchor domains (*green*), and central transporter (*light green*). The comparison between the two panels illustrates the power of integrative structural biology to provide detailed descriptions of complex biomolecular systems. NPC, Nuclear Pore Complex.

The integrative modeling workflow iterates through four stages that convert input information into an output model (Fig. 2): (i) gathering all available experimental data and prior information (physical theories, statistical analyses, and other prior models); (ii) translating information into representations of model components and a scoring function for ranking alternative models; (iii) sampling models; and (iv) validating the model. In this four-stage scheme, input information can contribute toward a model in five different ways, guided by maximizing the accuracy and precision of the model while remaining computationally feasible: (i) representing components of a model with some variables; (ii) scoring a model for its consistency with input information; (iii) searching for good-scoring (acceptable) models; (iv) filtering models based on the input information; and (v) validating the resulting models. We now discuss each of these ways in turn, using integrative structure modeling of the yeast NPC as an example (Fig. 3) (4).

### Nuclear pore complex

The yeast NPC is a ∼52-MDa complex that consists of ∼550 protein subunits of ∼30 different types. It is embedded in the nuclear envelope and plays a central role in cell biology by mediating nucleocytoplasmic transport of proteins and RNA, *via* cognate transport factors. The sheer size and flexibility of the NPC makes it all but impossible to solve its molecular architecture by conventional atomic resolution techniques, such as X-ray crystallography. However, integrating information from multiple sources, including stoichiometry from protein quantification, residue distances from chemical cross-linking with mass spectrometry, and the overall NPC shape from cryoelectron tomography, resulted in a relatively precise model.

### Model representation

First, input information can be used to define model representation (5). The representation specifies the variables whose values will be determined by modeling. Thus, it specifies the components of the system, such as atoms, coarse-grained particles, and subunits in a complex, including their copy numbers. It also specifies the type of component coordinates, such as positions, orientations, and conformations, and potentially other aspects of a model (below). For example, in crystallography, model representation commonly includes Cartesian coordinates and an isotropic temperature factor for each atom in the molecule. In the NPC modeling, quantitative MS and *in vivo* calibrated imaging determined the copy numbers of the constituent NPC proteins. The NPC model was represented by the positions and orientations of its parts. These parts included (i) rigid bodies corresponding to individual protein domains, subunits, or even subcomplexes for which the structure was determined previously by crystallography, comparative protein structure modeling, or integrative modeling and (ii) flexible regions otherwise. Representations were coarse-grained by using spherical beads corresponding to multiple amino acid residues to reflect the lack of information and/or to increase the efficiency of searching for acceptable models.

### Scoring function

Second, information can be used to construct a scoring function and compute its value. The scoring function quantifies the degree of a match between a tested model and the input information. The most common scoring function is a weighted sum of spatial restraints; each restraint is a function of the deviation of the computed property of a model from its measurement. An acceptable model is a model that sufficiently satisfies input information by some definition. Spatial restraints on the NPC structure included a correlation coefficient between a model and a cryoelectron tomography map of the entire complex at 28-Å resolution, 3077 distance restraints on pairs of residues spanning chemical cross-links, the rigid shape of the nuclear envelope pore, an excluded volume penalty, and others.

### Searching for models

Third, information can be used to constrain the model search space. Although rarely computationally feasible, the best search is a systematic enumeration of a defined search space, going through every possible model one by one with sufficient granularity (29). In practice, other methods, such as stochastic sampling *via* a Monte Carlo scheme (30), are often used. The demands on sampling increase with the number of degrees of freedom spanning the model, which in turn depends on the size of the modeled system and the detail with which it is represented. Sampling efficiency also depends crucially on the shape of the scoring landscape (cf, for many samplers, a funnel landscape is easier to sample than a golf course landscape) and the efficiency of evaluating the scoring function for a given model. The search for acceptable structural models of the NPC relied on replica exchange Gibbs sampling, based on the Metropolis Monte Carlo algorithm (31, 32, 33), starting with random initial structures in the context of the nuclear envelope. In addition, to increase the efficiency of sampling, only one of the eight symmetry units of the NPC was sampled, while still evaluating the scoring function for the complete NPC *via* appropriate consideration of its C_8_-symmetry. Hundreds of thousands of independent Monte Carlo runs resulted in a set of similar models that sufficiently satisfied the input information.

### Filtering models

Fourth, some information can be used for filtering acceptable models after they are produced by searching. Such use is often the case for information that is computationally expensive to incorporate into a scoring function, which is commonly evaluated thousands or millions of times during sampling. An example is using a negative-stain EM 2D class of a complex to find all those molecular docking solutions whose 2D projections match the class (33, 34, 35, 36). Integrative modeling of the NPC did not rely on any filtering.

### Validating models

Finally, a subset of information can be set aside to validate a model. Validation of a model is essential to avoid its overinterpretation (21). Just like scoring and filtering, validation also depends on assessing a degree of consistency between a model and some information not used to compute the model. The NPC model was tested, for example, by comparison with previously published lower-resolution data, including affinity copurifications and immuno-EM localizations of tagged protein components (17). In addition to assessing the acceptable models by comparison against information not used for modeling, the validation also includes an assessment of the thoroughness of structural sampling (sampling precision (37)), quantification of the agreement between the model and information used for modeling, and estimation of model precision. The model precision is defined by the variability among the acceptable models, provided sufficient sampling was performed (9, 37). In fact, it is this set of superposed acceptable models that can be considered the final model, equivalently to the ensemble of structures computed based on NMR data. The yeast NPC map localizes the 452 constituent proteins with an average precision of ∼1 nm. Independently determined NPC structures are in broad agreement with each other (4, 38, 39, 40, 41, 42, 43), with differences between the models resulting from the heterogeneity of the NPC architecture, different conditions used to purify and characterize physical samples, variation among the species, and uncertainty in the models themselves.

### Workflow iteration

The entire four-stage modeling process is generally performed iteratively until a satisfactory model is obtained. In the early iterations, modeling can pinpoint inconsistencies in the input data and modeling assumptions. For example, it may not be possible to find a model of an assembly of multiple components that satisfies reliable chemical cross-link restraints for given component copy numbers, prompting a re-examination of stoichiometry measurements (44). Similarly, the satisfaction of available data may require using a flexible instead of a rigid representation of subunits in a complex (45). Another example is the validation of a crystallographic interface between two components observed in a crystallographic study of the dimer, by its approximate reproduction in a model based on less precise data (35); this validation can then be followed up by imposing the validated crystallographic interface as a constraint to obtain a more precise model. Thus, integrative modeling can contribute continually during a combined experimental and computational study of a system and is best initiated early in a structure determination effort. Integrative studies are distinctly multidisciplinary and consequently often require a team of collaborators, including both experimentalists and modelers.

### Using integrative structures

An integrative approach frequently results in a coarse model depicting the molecular architecture instead of a detailed atomic structure. Nevertheless, such lower-resolution depictions still have many applications (5). For example, the NPC map has revealed insights about its architecture, function, and evolution (4, 34, 46, 47). It is also conceivable that the structure will facilitate studying how the NPC assembles and disassembles during the cell cycle; how it interacts with other key systems in the cell, such as chromatin and the spindle pole body; and how its function can be modulated to study the basic biology of the cell as well as for therapeutic purposes.

## Software for integrative modeling

**Figure 4:**
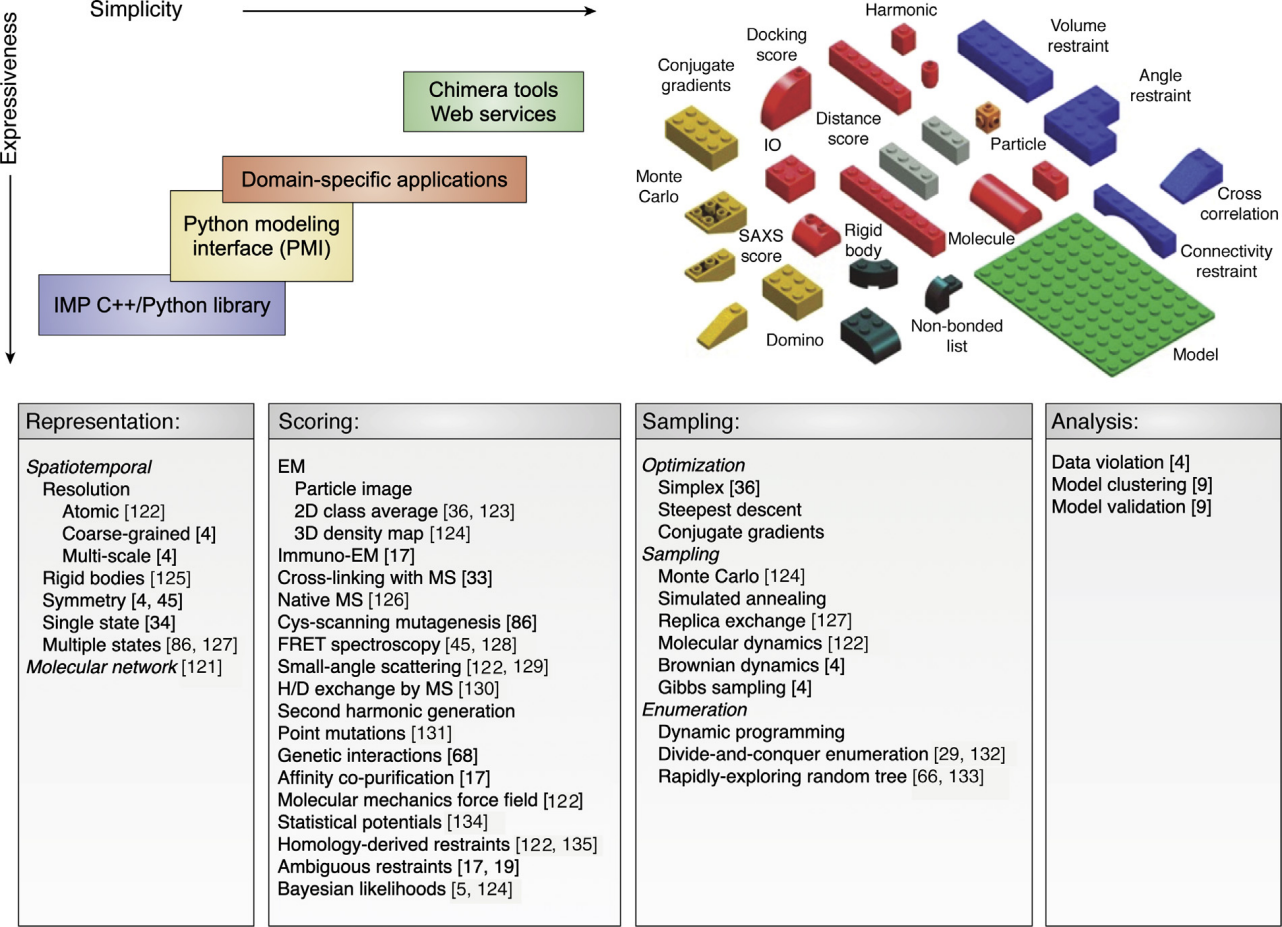
**IMP architecture.***Top*, IMP has a multitiered architecture, with the kernel written in C++, with progressively simpler levels that facilitate easier access to IMP functionality, but at a cost of losing some flexibility. The simplest access to IMP is enabled through its interfaces to web servers and to the molecular visualization program ChimeraX (71, 120). A more flexible layer is the *Python Modeling Interface* (PMI) that allows users to script the representation, scoring, sampling, and analysis protocols in Python (62). The Lego blocks indicate the modularity of IMP and the ability to mix and match different model representations, scoring functions, and sampling methods. *Bottom*, IMP implements various model representations, scoring function terms, sampling schemes, and analyses; publications exemplifying the use of various functionalities are cited. The model can be represented at different resolutions, with some parts rigid while others are flexible. A model can also specify multiple states of the system as well as a single state. In addition to spatiotemporal models, IMP has been used to compute models of molecular networks by satisfaction of network restraints (121). The agreement between input information and a model is quantified by a scoring function, which is constructed with the aid of a library of different functional forms (*e.g.*, harmonic and harmonic upper bound) that can operate on different spatial features of the modeled system (*e.g.*, positions, distances, and shape), based on a variety of input information. In ambiguous restraints, the assignment of a restraint to specific particles can itself be a variable, as is needed for chemical cross-links in a system with multiple copies of the cross-linked protein. Increasingly, the scoring function terms correspond to Bayesian data likelihoods. Data-based restraints can be supplemented by a molecular mechanics force field, homology-derived restraints, and various statistical potentials. A number of schemes for searching for good-scoring models are available, including local refinement methods and stochastic sampling methods in the continuous space of Cartesian coordinates as well as enumeration schemes in the discrete spaces of both Cartesian and internal coordinates. Finally, IMP implements protocols for analyzing and validating models, including methods for model clustering based on their structural similarity (4, 5, 9, 17, 19, 29, 33, 34, 36, 45, 66, 68, 86, 121, 122, 123, 124, 125, 126, 127, 128, 129, 130, 131, 131, 132, 132, 133, 134, 135). IMP, *Integrative Modeling Platform*.

A number of computer programs have been used for integrative structure modeling, including HADDOCK (48, 49), Rosetta (50, 51), PHENIX (52), BCL (53), XPLOR-NIH (54), TADbit (55, 56), PGS (57), iSPOT (58), FPS (59), PatchDock (60), BioEn (61), and others (5, 10). Next, we review our own open-source IMP software package that aims to provide comprehensive support for implementing and distributing integrative modeling protocols (Fig. 4) (https://integrativemodeling.org) (8, 62). IMP's relative strengths include a large variety of model representations, scoring functions based on different data, and sampling schemes, all of which can be mixed and matched relatively easily with each other to facilitate integrative structure modeling. Another distinction is an increasingly Bayesian perspective on uncertainties in input information, model representations, and scoring functions.

### Integrative Modeling Platform

The development of IMP has been guided by the requirement for general, flexible, and modular software for integrative modeling. To maximize the applicability of integrative modeling to new problems, the software needs to be readily available, well documented, supported, and easy to learn. IMP was designed to appeal to both method developers, who can contribute their own IMP modules while benefiting from the rest of IMP, and users, who prefer simple access to the existing IMP functionality. A key design feature of IMP is that it allows mixing and matching of different model representations, scoring functions, and model sampling schemes, followed by executing various protocols for model validation and analysis. This modularity has greatly facilitated our application of IMP to difficult modeling problems, where multiple iterations through defining model representation and spatial restraints, performing structural sampling, and analyzing the results are needed. IMP has been used mainly for computing architectures of macromolecular complexes by assembling subunits of known structure based on 3DEM maps and chemical cross-links, as exemplified by the integrative structure determination of the NPC (above).

## Challenges in integrative modeling

There are a number of interrelated challenges in improving integrative modeling. Addressing these challenges will improve the accuracy and precision of integrative models as well as expand the types of systems that can be modeled to include larger and more complex, heterogeneous, and dynamic systems. We briefly outline several challenges as follows.

### Optimizing model representation

In difficult modeling cases, model representation is the first and possibly most important modeling decision. The choice of model representation generally balances the accuracy of translating input information into restraints on models with the efficiency of sampling alternative models (*e.g.*, rigid *versus* flexible subunit structure, single *versus* multiple state models). For example, when only chemical cross-links between pairs of residues are available to guide the fitting of subunits into a medium-resolution 3DEM map, it is often sufficient to represent the individual subunits as rigid bodies at a single residue-per-bead resolution, as opposed to flexible structures at atomic resolution. The representation is generally selected ad hoc, based on experience. Therefore, objective methods for selecting an optimal representation given available input information and sampling power are needed (63). Such methods could in principle be guided by Bayesian model selection or even Bayesian model averaging (64, 65).

### Expanding the variety of model representations

Model representation determines the type of model that is computed. Thus, expanding the set of possible representations of integrative models expands the scope of integrative modeling. Currently, atomic and various coarse-grained models of systems in single and a few discrete static states can be produced relatively routinely (Fig. 3). In contrast, much work remains to be done to compute integrative models of heterogeneous systems existing in many states (61, 66). Similarly unchartered is integrative modeling of spatiotemporal processes, so that the resulting spatiotemporal models are by construction consistent with available experimental data (4, 47). In contrast, various flavors of molecular dynamics simulations compute models based on physical principles alone, followed by validation of the resulting trajectories based on experimental data only after simulation. Additional opportunities for expansion of integrative modeling include modeling of molecular and other types of networks (*e.g.*, metabolic and signaling networks), energy landscapes (defining structures, thermodynamics, and kinetics), reaction fluxes, and diffusion processes. For systems whose salient features include both structural and network degrees of freedom, such as spatially organized metabolic networks (*e.g.*, polyketide synthases (67)), it may be especially attractive to couple structural and network variables into a single unified representation informed by both structural and network data, thus hopefully maximizing the accuracy, precision, and completeness of the resulting models.

### Expanding the variety of input data and prior information

Increasing the variety of input data and prior information used for modeling will expand the scope of structural biology. For example, an upper bound on a distance between two point mutations in a protein complex can be inferred from quantitative genetic interaction mapping of point mutations in the background of gene knockouts (68). Such data can be obtained by measuring the size of yeast colonies across a set of mutant strains, therefore allowing structure characterization without purifying a sample for traditional structural biology methods. Likewise, protocols need to be developed for including into integrative modeling other types of data, such as electron and X-ray tomography maps, *in vivo* chemical cross-links, and super-resolution optical microscopy images. Including some of these data may require developing new model representations first.

### Bayesian scoring function

Quantifying a match between input information and a model in a fully Bayesian fashion increases the accuracy and optimizes the precision of the resulting models (31). A Bayesian posterior model density is proportional to the individual data likelihoods and priors (5, 69). Data likelihood quantifies the probability of observing the data given the model (or some of its aspects), whereas a prior quantifies the prior probability of the model (or some of its aspects). Thus, a Bayesian scoring function also facilitates the modularity of integrative modeling development, as different data likelihoods and priors for separate sources of information can be independently developed by experts in relevant fields and then simply multiplied to obtain the complete posterior model density.

### Model sampling

It is necessary that all models consistent with input information are found, not only a subset of them (*i.e.*, overfitting must be avoided) (37). Sufficient sampling, as opposed to optimization, is especially required for analyzing a Bayesian posterior model density (70). If a sufficient model sample is in hand, the uncertainty of a model can be directly estimated based on the variability in the sample (9). Thus, efficient structural sampling algorithms and computing hardware are needed.

### Model analysis

Once a model is in hand, it needs to be validated and interpreted. Although rudimentary protocols for estimating the uncertainty of integrative structure models do exist (4), it is desirable that not only the scoring function but also the validation of an integrative structure is formulated in a Bayesian fashion (10, 11). Of importance, such a formulation may facilitate deconvoluting total model uncertainty into uncertainty arising from lack of information *versus* actual heterogeneity in the samples used to collect the data. In addition, interpretation of any model, and especially a structural model, is generally facilitated by its visualization. Because the representation of integrative structures is often nonatomic, new visualization programs, such as ChimeraX (71) and Mol∗ (72), were developed. These and other programs need to be continually updated to be able to handle the growing variety of integrative models and the data on which they are based.

## Integrative structural biology community is nucleated by the wwPDB

### Protein Data Bank

In a visionary achievement, the PDB was founded in 1971 when few protein structures were available (73), followed by the creation of a federation of the US, European, and Japanese sites in 2003 (7). Ever since its founding, the PDB has catalyzed and shaped structural biology. The impact of a freely accessible and comprehensive repository for all published biomolecular structures and corresponding experimental data cannot be overstated. In addition to archival and dissemination, wwPDB has also established *de facto* standards for validation and publication of X-ray (74), NMR (75), 3DEM (76), SAS (77, 78), and integrative structures and data (10, 11) as well as comparative protein structure models (79, 80). In the process, the PDB has vastly improved the efficiency, quality, and scope of structural biology. For example, it is inconceivable to imagine the field of structural bioinformatics without the PDB. The PDB inspired many new methods for analysis and prediction of protein structures, provided input data for these methods, and thus facilitated the discovery of numerous protein sequence–structure–function principles; for example, the PDB provides inputs for sequence and structure comparison, studying the impact of sequence and structure changes on function, computing sidechain rotamer libraries and other statistical potentials, threading sequences through structures, comparative modeling, molecular docking, and integrative modeling. In a positive feedback loop, some of these advancements, in turn, facilitated improving methods for structure determination, contributing to additional growth of the PDB and increased accuracy of biomolecular structures. Moreover, the impact of the PDB extends beyond structural biology, as it facilitates using structural information in other fields as well, including perhaps most importantly in cell biology and drug discovery (81). Finally, the PDB is a beacon for community-building efforts in any field, clearly illustrating the beneficial impact of a freely accessible and comprehensive resource for the data and models defining the field. Indeed, our vision for mapping the cell is based in part on this insight (below).

### PDB for integrative modeling

The wwPDB has played several important roles in the development of the integrative structural biology community. The leadership of wwPDB organized and catalyzed meetings of scientists interested in various aspects of integrative structural biology, focusing on representation, validation, archival, and dissemination of integrative structure models and data (10, 11). At the first meeting in 2014, the participants agreed to establish an Integrative/Hybrid Methods (IHM) Task Force to work on a common set of evolving standards (11). Recommendations on several key issues were made, followed by their implementation that culminated in the nascent PDB-Development (PDB-Dev) archive for integrative structures and data (https://pdb-dev.wwpdb.org) (82, 83). PDB-Dev is a standalone prototype system for collecting, curating, validating, archiving, and disseminating integrative structures and data. To facilitate an agile development platform, PDB-Dev is implemented separately from the PDB, with the plan to integrate it into PDB in the coming years. This integration will be achieved by making the wwPDB OneDep deposition system and other aspects of the PDB data pipeline (84) fully supportive of integrative structures and data. PDB-Dev is enabled by an expanded set of data standards for representing integrative structures and data; a software library that supports these data standards; a data harvesting system for collecting heterogeneous data from diverse experimental techniques; methods for curating, validating, and visualizing integrative structures; and web services for distributing archived data. Through the PDB-Dev website, scientists interested in integrative structures can search and retrieve archived structures, visualize multiscale and multistate structures, gather information regarding the input data and methods used in modeling, and download the archived data for further research. PDB-Dev already contains 55 entries as of February 1, 2021, even though the number of depositions has not been maximized during the current development stage. Integrative structures can be presently deposited only in PDB-Dev, not PDB. One reason is that PDB-Dev offers a much richer set of model representations than the standard atomic representation used by the PDB. The editors of scientific journals are increasingly requesting that authors deposit their integrative structures and data into PDB-Dev, for the benefit of the authors and the community. Next, we discuss the representation, validation, and archival of integrative structures in PDB-Dev in more detail.

### Model representation

Traditional structural biology methods usually produce a single atomic coordinate set. In contrast, integrative models tend to be more complex in at least four respects (11). First, a model can be multiscale, coarse-graining different levels of structural detail by a collection of geometrical primitives (*e.g.*, points, spheres, tubes, Gaussians, and probability densities) (85). Thus, the same part of the system can be described with multiple representations and different parts of the system can be represented differently. Second, a model can be multistate, specifying multiple discrete states of the system that are needed simultaneously to explain the input information (each state might differ in structure and/or composition) (86, 87). Third, a model can also specify the order of states. This feature allows a representation of a multistep biological process, a functional cycle (88), a kinetic network (89), or a time evolution of a modeled system (*e.g.*, a molecular dynamics trajectory) (90). Finally, an ensemble of models is often provided to specify the uncertainty in the input information by including each model that on its own satisfies the input information within an acceptable threshold. This aspect of the representation allows us to describe model uncertainty resulting from the incompleteness of input information; such ensembles are distinct from multiple states that represent actual variations in the structure, as implied by experimental information that cannot be accounted for by a single representative structure (21, 28). Thus, the generalized representation allows us to encode an ensemble of multiscale, multistate, and ordered models (82, 83). This expanded molecular representation is implemented *via* an extension of the PDBx/mmCIF dictionary (82, 91).

### Model validation

Assessment of both an integrative structure and the data on which it is based is of critical importance for guiding structural interpretation. This assessment is a major research challenge owing to the diverse types of input experimental data and computational methods used in integrative modeling. Correspondingly, the IHM Task Force put forward recommendations for creating methods to validate integrative structures (10, 11). There are currently four main categories of assessment: (i) quantifying the quality of experimental data used to compute and/or asses an integrative structure; (ii) application of extant PDB criteria to assess atomic integrative structures (74); (iii) evaluating the fit of a structure to experimental data and other information, whether or not this information was used to compute the structure; and (iv) estimating the uncertainty (precision) of the structure. These recommendations are being pursued by the PDB-Dev team, with the benefit of contributions from members of the wwPDB IHM Task Force and others. For example, proposed experimental data quality criteria are based on the respective community practices (92, 93, 94) and a number of model validation criteria are taken from IMP (4, 9). The validation pipeline will also leverage existing software developed by the structural biology community (*e.g.*, wwPDB (95), MolProbity (96), BMRB (97), EMDB (98, 99, 100), SASBDB (101), PHENIX (52), and PDBStat (102)). Standardized validation of integrative structures will ultimately be part of deposition into the PDB, as is already the case for structures derived using traditional methods (74, 75, 76, 77, 78, 95). The validation report will facilitate reviewing, publishing, disseminating, and using the results of integrative structural biology studies.

### Federated archive

**Figure 5:**
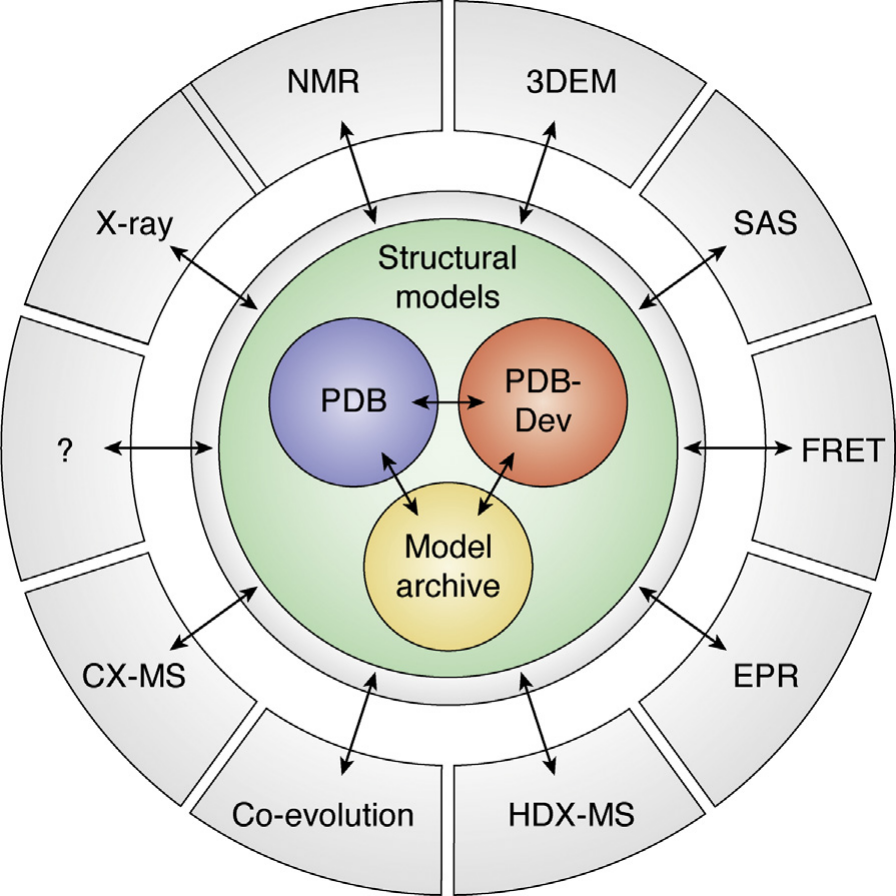
**Federated wwPDB archive for integrative structures and data.** The scheme (courtesy of Helen Berman) shows the current organization of the archive. The atomic structures produced by traditional methods are stored in the PDB, integrative structures in the PDB-Dev, and comparative protein structure models in the Model Archive (79). The data on which the structures are based are stored in separate databases, often constructed by experts in the community that is generating the data. All resources are interlinked with each other. Many additional data archives are expected to be added in the future, reflecting and catalyzing progress in integrative structural biology. 3DEM, three-dimensional electron microscopy; EPR, electron paramagnetic resonance; FRET, Foerster resonance energy transfer; PDB-Dev, PDB-Development; SAS, small-angle scattering; wwPDB, Worldwide Protein Data Bank.

To support the archival, validation, and dissemination of integrative structures and data, wwPDB initiated a federated network of interoperating structural biology data resources (Fig. 5) (103, 104), as recommended by the IHM Task Force (10, 11). This effort involves collaboration with teams working on other repositories of experimental data, such as BMRB for NMR data (97), EMDB for EM data (98), SASBDB for SAS data (101), and PRIDE for MS data (105). The goal is to create standards and tools for automated data exchange between the PDB and member repositories. Discussions with experts in additional methods, including Foerster resonance energy transfer spectroscopy and microscopy (106), hydrogen/deuterium exchange by MS, and genome modeling, are in progress. More are expected in the future. Thus, integrative structural biology efforts are expanding the structural biology community by connecting it with other communities generating the types of data not previously used for determining protein structures. Conversely, these other communities stand to benefit more directly from structural biology than has been the case so far. In a reductionist view of cell biology, biomolecular structures underlie all systems and processes studied in cell biology. Thus, it is fitting that a structural archive nucleates connections with archives that store other types of data collected in cell biology. The federated wwPDB archive will advance scientific research by promoting efficient data sharing and making research data easily accessible to everyone, impacting both structural biologists and users of structural biology data, including cell biologists and others.

### Software development

wwPDB may also play an important role in the development of software for integrative modeling in at least two ways. First, wwPDB could provide benchmarks for new modeling methods, including experimental datasets, reference structures, and criteria for assessing the quality of the methods, perhaps in conjunction with other community benchmarking efforts, such as EM Challenge (http://challenges.emdatabank.org/?q=model_challenge) and Critical Assessment of Protein StructurePrediction (107). Second, wwPDB could catalyze standard interfaces between key molecular modeling operations. Such standards would facilitate mixing and matching of functionalities across molecular modeling programs, not only within one program (*cf.*, Fig. 4). As a result, the efficiency, quality, and scope of structural studies would be improved.

## Taking integrative modeling to the next level: Metamodeling of the cell

### Cell modeling

Modeling the cell (108) is one of the grand challenges in cell biology (12). Most of the current cell modeling approaches rely on a single type of representation of the cell; for example, spatiotemporal (109), ordinary differential equation (110), and flux balance analysis representations (111). In addition to cell models, there are a myriad of models of different parts of the cell, too numerous to review here. These models may provide a useful starting point for cell modeling, owing to their encoding of expertise, data, and computing used to produce them. However, no general approach yet exists for combining different kinds of models, although steps in this direction have been made (112, 113, 114, 115).

### Cell model

Despite these efforts, it is not yet clear what a cell model should look like. More precisely, it is not yet clear what the representation of the cell model should be. Nevertheless, it is likely that such a model will need to have a number of desirable attributes, as follows. First, the cell is organized spatially and changes with time; thus, the cell model should be spatiotemporal, defining the positions of its parts in space and time. Second, the cell is modular and hierarchical, with the hierarchy spanning atoms, molecules, organelles, and the cell; thus, the model should be multiscale, including all representations that are useful for reflecting available information onto the model and for interpreting the model, including nonspatial representations such as molecular networks represented by graphs. Third, the depictions of the cell using these varied representations should clearly be in harmony with each other; for example, when two molecules are interacting in a molecular network representation of a signaling pathway in the cytosol, they should also have sufficient propensity to be in proximity of each other in a Brownian dynamics simulation of the cytosol and *vice versa*. Fourth, the model needs to be as useful as possible; thus, it needs to be maximally accurate, precise, complete, and general. Fifth, the cell is a complex object with many parts and degrees of freedom, requiring a large amount of information to specify; thus, the model should be integrative, based on all available information. Sixth, the model will be imprecise; thus, its uncertainty should be specified. Seventh, in addition to being able to rationalize known facts, the model should also be testable and useful in guiding future experiments; thus, it should allow predicting outcomes of future experiments. Finally, the model will require a large community effort on data collection over a long period of time, in turn requiring iterative and objective improvement of the model; thus, it should be computed from input information automatically and efficiently. A model with all these attributes would clearly be of immense value to both cell biology and drug discovery.

### Integrative modeling for cell mapping

It is tempting to adopt integrative modeling for cell modeling. In a brute force approach, model representation would be expanded to include all degrees of freedom of interest, informed by all experimental data and prior information. In practice, however, such a generalization is clearly unlikely to succeed any time soon, owing to insufficient data, prior information, and computing power as well as limitations of existing integrative modeling methods. Thus, substantive development of integrative modeling is needed. We outline one such recent development here, called metamodeling (13).

### Metamodeling

**Figure 6:**
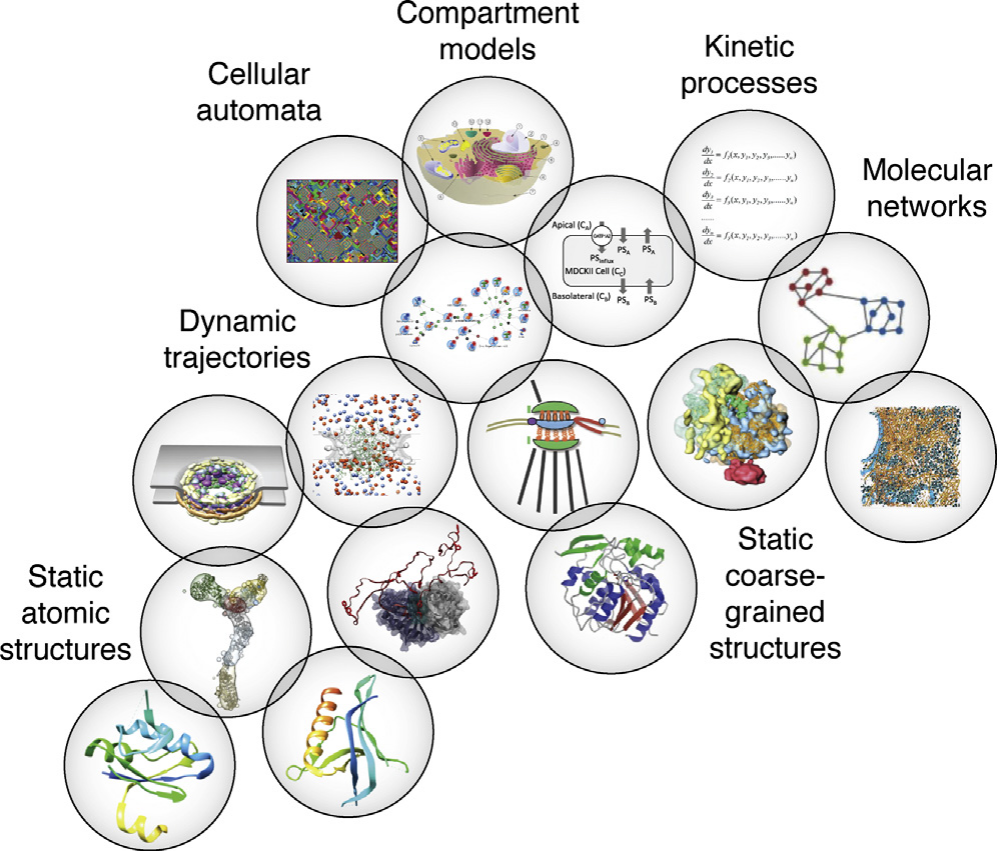
**Metamodeling.** The scheme conveys the aim of metamodeling, which is to combine input models of different types (*circles*) to obtain a metamodel. The process involves harmonizing input models with each other, as indicated by the overlaps between the circles. Different types of models shown in the scheme include static structures of biomolecules at atomic and coarse-grained resolutions, dynamics of biomolecules represented by molecular dynamics trajectories, processes involving biomolecules represented by Brownian dynamics trajectories, molecular networks represented by graphs, density maps, diffusion, kinetic processes quantified by systems of ordinary differential equations, a cellular automaton, and a compartment model of the cell. The coupling between models, which allows for their harmonization, is indicated by overlapping *circles*.

Metamodeling is a divide-and-conquer modeling approach that aims to integrate varied input models into a metamodel (Fig. 6). Thus, metamodeling can be seen as a special case of integrative modeling in which the focus is on integrating prior models instead of data. The large problem of computing an integrative model of the cell is broken into a number of smaller modeling problems corresponding to computing models of some aspects of some parts of the cell. Each such input model may be informed by different subsets of available data, relying on its distinct model representation at any scale and level of granularity. Metamodeling then proceeds by assembling and harmonizing the input models into a complete map of the cell. A simple example of metamodeling is the flexible docking of two protein structures into a complex. In this example, the input models are the two apo structures that are refined and juxtaposed by docking (metamodeling) to model the induced fit and the final binding mode (metamodel). Although combining the two input models in this special case is familiar, combining models of different types is generally challenging.

### Bayesian metamodeling

Bayesian metamodeling is a specific implementation of metamodeling in which input models are harmonized through a Bayesian statistical model of their relations with each other and/or the physical world. This Bayesian approach enables us to update our “beliefs” in the distribution of model variables (including best single-value estimates and their uncertainties), given information provided by all input models. Bayesian metamodeling proceeds through the following three stages: the input models are (i) converted to a standardized statistical representation relying on Probabilistic Graphical Models, (ii) coupled by modeling their mutual relations with the physical world, and (iii) finally harmonized with respect to each other *via* backpropagation. Bayesian metamodeling was illustrated by a proof-of-principle metamodel of glucose-stimulated insulin secretion by human pancreatic ß-cells. The input models included a coarse-grained spatiotemporal simulation of insulin vesicle trafficking, docking, and exocytosis; a molecular network model of glucose-stimulated insulin secretion signaling; a network model of insulin metabolism; a structural model of glucagon-like peptide-1 receptor activation; a linear model of a pancreatic cell population; and ordinary differential equations for systemic postprandial insulin response. The coupling of the input models increased their accuracy and reproduced the behavior of the ß-cell not represented by any individual input model, such as the incretin effect.

### Metamodeling facilitates community collaboration

**Figure 7:**
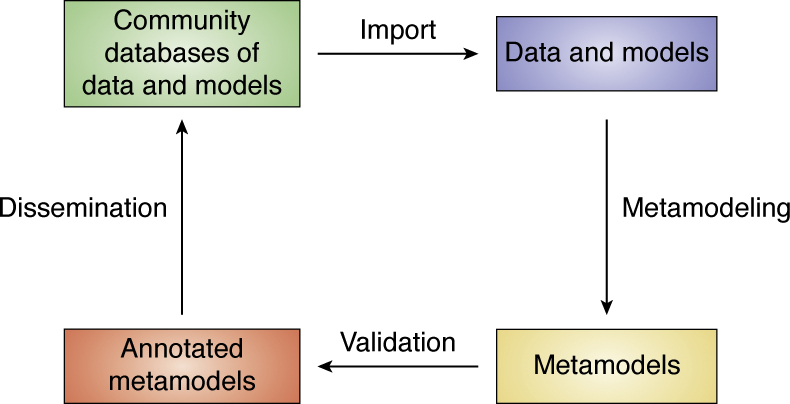
**Vision for the contribution of metamodeling to mapping the cell.** The scheme illustrates how iterative metamodeling could contribute toward the mapping of the cell.

Metamodeling benefits from decentralized computing, while often producing a more accurate, precise, and complete model that contextualizes input models as well as resolves conflicting information. By shifting the focus from data integration to model integration, metamodeling facilitates the sharing of data, computational resources, expertise in diverse fields, and already existing models of the cell and its parts. Thus, by construction, metamodeling may be more useful for the large effort of cell mapping than centralized approaches to data integration. At its core, metamodeling is rooted in collaboration and appreciation for the details of disparate data, methods, and models, which cannot be achieved by any individual scientist, research group, or institution. Acting on this premise, the Pancreatic ß-Cell Consortium (12) is creating cyberinfrastructure for archiving and disseminating experimental data and models that will include metamodeling (Fig. 7). Thus, each time a data set is determined or a model is computed, the metamodel can be updated automatically. Together with the many community databases of data and models (7, 111, 116, 117, 118), metamodeling may help create an effective sociotechnical ecosystem that is no doubt required for successful mapping of the cell.

## Conclusions

Integrative modeling is a general approach for computing any kind of a model based on varied types of data and prior information. It can already produce structures of biomolecules that are recalcitrant to traditional structural biology methods, as exemplified by the integrative structure of the NPC. Moreover, its applicability to large, complex, and dynamic systems is increasing, as current shortcomings are being addressed. wwPDB activities, including leadership and service, are contributing significantly to the integrative structural biology community. For example, wwPDB maximizes the impact of integrative structural biology on cell biology by archiving and disseminating integrative structures and the data on which they are based. A special case of integrative modeling, called metamodeling, aims to combine multiple types of models instead of multiple datasets into an output model, potentially providing a practical means toward mapping the entire cell. With the progress of integrative structural biology, many more cell biologists will also become structural biologists, for their own gain.

## Conflict of interest

The author declares that he has no conflicts of interest with the contents of this article.
